# Classifying chest CT images as COVID-19 positive/negative using a convolutional neural network ensemble model and uniform experimental design method

**DOI:** 10.1186/s12859-021-04083-x

**Published:** 2021-11-08

**Authors:** Yao-Mei Chen, Yenming J. Chen, Wen-Hsien Ho, Jinn-Tsong Tsai

**Affiliations:** 1 School of Nursing, Kaohsiung Medical University, Kaohsiung, 807 Taiwan; 2 Superintendent Office, Kaohsiung Medical University Hospital, Kaohsiung, 807 Taiwan; 3 Management School, National Kaohsiung University of Science and Technology, Kaohsiung, 824 Taiwan; 4 Department of Healthcare Administration and Medical Informatics, Kaohsiung Medical University, Kaohsiung, 807 Taiwan; 5 Department of Medical Research, Kaohsiung Medical University Hospital, Kaohsiung, 807 Taiwan; 6 Department of Computer Science, National Pingtung University, Pingtung, 900 Taiwan

**Keywords:** COVID-19, Chest computed tomography image, Convolutional neural network, Algorithm hyperparameter, Ensemble model

## Abstract

**Background:**

To classify chest computed tomography (CT) images as positive or negative for coronavirus disease 2019 (COVID-19) quickly and accurately, researchers attempted to develop effective models by using medical images.

**Results:**

A convolutional neural network (CNN) ensemble model was developed for classifying chest CT images as positive or negative for COVID-19. To classify chest CT images acquired from COVID-19 patients, the proposed COVID19-CNN ensemble model combines the use of multiple trained CNN models with a majority voting strategy. The CNN models were trained to classify chest CT images by transfer learning from well-known pre-trained CNN models and by applying their algorithm hyperparameters as appropriate. The combination of algorithm hyperparameters for a pre-trained CNN model was determined by uniform experimental design. The chest CT images (405 from COVID-19 patients and 397 from healthy patients) used for training and performance testing of the COVID19-CNN ensemble model were obtained from an earlier study by Hu in 2020. Experiments showed that, the COVID19-CNN ensemble model achieved 96.7% accuracy in classifying CT images as COVID-19 positive or negative, which was superior to the accuracies obtained by the individual trained CNN models. Other performance measures (i.e., precision, recall, specificity, and F_1_-score) obtained bythe COVID19-CNN ensemble model were higher than those obtained by individual trained CNN models.

**Conclusions:**

The COVID19-CNN ensemble model had superior accuracy and excellent capability in classifying chest CT images as COVID-19 positive or negative.

## Background

The rapid spread of coronavirus disease 2019 (COVID-19) since the beginning of 2020 has often exceeded the capability of doctors and hospitals in many regions of the world. One effective tool for detecting COVID-19 is chest computed tomography (CT). Although a CT scan can be performed in several minutes, the time needed for a radiologist to review and classify the image is much longer. Therefore, tools for automatically detecting or diagnosing COVID-19 are extremely valuable and urgently needed.

### Literature review

Gozes et al. [1] developed automated CT image analysis tools that used a Resnet-50 deep convolutional neural network (CNN) to detect coronavirus and to quantify the burden on healthcare systems. The study reported that deep-learning image analysis of thoracic CT images achieved 98.2% sensitivity, 92.2% specificity, and 0.996 area under curve (AUC) in classifying results as positive or negative for coronavirus.Another COVID-19 diagnosis method developed in Hu et al. [2] used a CNN with a ShuffleNet-v2 backbone to distinguish between CT images of patients with and without COVID-19 infection. Their experimental results indicated that the diagnostic model was accurate not only in identifying COVID-19, but also in distinguishing between COVID-19 infections from other viral infections. Li et al. [3] developed a COVNet framework using Resnet-50 as the backbone that detected COVID-19 by using a neural network to extract visual features from volumetric chest CT exams. In independent testing, per-exam sensitivity in detecting COVID-19 was 90% (114 of 127), and per-exam specificity was 96% (294 of 307). Shan et al. [4] developed a modified 3-D convolutional neural network that combines V-Net with the bottle-neck structure for automatically segmenting and quantifying infected regions in CT scans of COVID-19 patients. Quantitative evaluations indicated that the system was highly accurate in automatically delineating infected regions. Song et al. [5] developed a CT diagnosis system that used a detailed relation extraction neural network to identify COVID-19 patients. According to their experimental results, the model identified COVID-19 infection with recall (sensitivity) of 0.93. Wang et al. [6] proposed a transfer learning neural network based on the Inception network that used chest CT images to screen for COVID-19. Internal validation tests revealed that the model had an overall accuracy of 89.5% with specificity of 0.88 and sensitivity of 0.87. In the external testing dataset, the model showed a total accuracy of 79.3% with specificity of 0.83 and sensitivity of 0.67. Xu et al. [7] established the Resnet-18 network with the location-attention mechanism that appeared promising for supplementary clinical use by frontline doctors in diagnosis and early screening of COVID-19 patients. Experiments performed using the benchmark dataset achieved 86.7% accuracy in screening CT images for COVID-19. In Yang et al. [8], the multi-task learning and self-supervised learning method of COVID-19 diagnosis based on CT images of COVID-19 achieved an F_1_-score of 0.90, an AUC of 0.98, and an accuracy of 0.89. According to the senior radiologist in that study, the models perform well enough for clinical use. According to the above literature on COVID-19 screening, most researchers have used a single model to classify chest CT images. Compared to an ensemble model in which classification is based on the results of the majority, however, a single model is more likely to make classification errors. Moreover, no studies have discussed how algorithm hyperparameters affect classification accuracy in a pre-trained CNN model. Therefore, further research is needed to improve classification accuracy.

### Motivation

The motivation of this study was to establish an ensemble model that uses majority voting strategy to screen chest CT images for COVID-19. In a pre-trained CNN model, learning speed and quality are determined by algorithm hyperparameters that are set before the learning process begins. In subsequent training, different pre-trained CNN models may require different algorithm hyperparameters (e.g., optimizer, learning rate, and mini-batch size) to improve their classification accuracy [9]. The current study used uniform experimental design (UED) to generate the combination of algorithm hyperparameters for a pre-trained CNN model. The experiments showed that the COVID19-CNN ensemble model had superior classification accuracy compared to a single model and excellent accuracy in classifying chest CT images as COVID-19 positive/negative.

### Problem description

Chest CT and X-Ray images are critical practical tools for diagnosis of COVID-19, because they can be used relatively quickly and easily to detect pneumonia-like symptoms of COVID-19. A recent study concluded that screening lung CT images is the best method of early-stage COVID-19 diagnosis and concluded that CT should be the primary screening method [10]. The severe respiratory symptoms of COVID-19 result in relatively high ICU admission and mortality rates in these patients. Manifestations of COVID-19 in CT images differ from those of other viruses that cause pneumonia, e.g., influenza-A [7]. Therefore, CT images have an untapped potential use in COVID-19 diagnosis.

During a COVID-19 outbreak, overworked radiologists may have limited time to review CT scans. Additionally, radiologists in rural and/or under-developed areas may not be adequately trained to screen CT scans for an emerging disease such as COVID-19. The considered problem was how to screen large numbers of chest CT images for COVID-19 efficiently and accurately. Since a CT showing evidence of COVID-19 is difficult to distinguish from a normal CT, machine learning may be a useful tool for assisting radiologists in screening CT images for COVID-19.

**Fig. 1:**
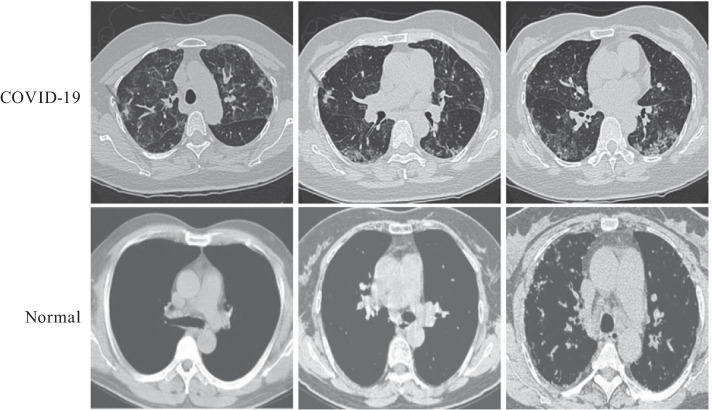
Representative CT images in the COVID-19 and Normal classes

The key slices of chest CT with suspected lesions were extracted from DICOM files by professional radiologists. All chest CT images used in the experiments in this study had been published previously [11]. The CT images were divided into two classes: COVID-19 and Normal. Figure 1 shows representative CT images in the two classes.

## Results

The proposed COVID19-CNN ensemble model integrated multiple trained CNN models for classifying chest CT images as COVID-19 positive or negative. The pre-trained CNN models included VGG-19, Resnet-101, DenseNet-201, Inception-v3, and Inception-ResNet-v2. The chest CT images obtained from COVID-19 patients in Hu [11] were used for training and performance validation of pre-trained CNN models. The testing set of chest CT images from COVID-19 patients was used for performance evaluation of the COVID19-CNN ensemble model. The experimental environment was Matlab R2019 with its toolboxes developed by MathWorks, and GPU GTX-1080Ti-11G.

The experimental data for chest CT images from COVID-19 patients included a training set, a validation set, and a testing set. To maintain compatibility with the CNN-based architecture and the developed software, each CT image was processed as a 224 × 224 × 3 image for the VGG-19, Resnet-101, and DenseNet-201 models or as a 299 × 299 × 3 image for the Inception-v3 and Inception-ResNet-v2 models, where 3 is the number of color channels. Table 1 shows the training, validation, and testing sets of chest CT images from COVID-19 patients.

**Table 1:** Training, validation, and testing sets of chest CT images from COVID-19 patients

Class	Training set	Validation set	Testing set	Total images
COVID-19	309	50	46	405
Normal	303	49	45	397
Total images	612	99	91	802

For training, different pre-trained CNN models require different algorithm hyperparameters that are set before the learning process begins. The algorithm hyperparameters for pre-trained CNN models in this study were ‘Optimizer’, ‘MiniBatchSize’, ‘MaxEpochs’, and ‘InitialLearnRate’. Optimizer was the training option. MiniBatchSize was a mini-batch at each iteration. MaxEpochs was the maximum number of training epochs. InitialLearnRate was an option for decreasing the learning rate during training.

The UED table of the minimum number of experiments for four factors is *U*_7_. Tables 2 and 3 show the seven-level uniform layout and selection table used for *U*_7_(7^6^), respectively. Table 4 shows that *U*_7_(7^4^) was selected from four factors in Table 3 and was used to design the combinations of the four algorithm hyperparameters for the seven levels*.* The levels for the ‘Optimizer’ hyperparameter were ‘adam (adaptive moment estimation)’ and ‘sgdm (stochastic gradient descent with a momentum)’. The values for the ‘MiniBatchSize’ hyperparameter ranged from 10 to 40. The values for the ‘MaxEpochs’ hyperparameter ranged from 4 to 10. The values for ‘InitialLearnRate’ hyperparameter were 10^–4^, 10^–5^, and 10^–6^. Table 5 shows the level values of the four algorithm hyperparameters for a pre-trained CNN model. Table 6 shows the seven combinations of the four algorithm hyperparameters that combined the values in Tables 4 and 5 and were used in a pre-trained CNN model for classifying chest CT images as COVID-19 positive or negative.

**Table 2:** Seven-level uniform layout of *U*_7_(7^6^)

Number of experiments	Number of columns					
	1	2	3	4	5	6
1	1	2	3	4	5	6
2	2	4	6	1	3	5
3	3	6	2	5	1	4
4	4	1	5	2	6	3
5	5	3	1	6	4	2
6	6	5	4	3	2	1
7	7	7	7	7	7	7

**Table 3:** Selection table used for *U*_7_(7^6^)

Number of factors	Number of columns
2	1 3
3	1 2 3
4	1 2 3 6

**Table 4:** Seven-level uniform layout of *U*_7_(7^4^) used to allocate four algorithm hyperparameters for seven levels

Number of experiments	Algorithm hyperparameters			
	Optimizer	MiniBatchSize	MaxEpochs	InitialLearnRate
1	1	2	3	6
2	2	4	6	5
3	3	6	2	4
4	4	1	5	3
5	5	3	1	2
6	6	5	4	1
7	7	7	7	7

**Table 5:** Level values of four algorithm hyperparameters for a pre-trained CNN model

Number of experiments	Algorithm hyperparameters			
	Optimizer	MiniBatchSize	MaxEpochs	InitialLearnRate
1	adam	10	4	10^–4^
2	sgdm	15	5	10^–5^
3	adam	20	6	10^–6^
4	sgdm	25	7	10^–4^
5	adam	30	8	10^–5^
6	sgdm	35	9	10^–6^
7	adam	40	10	10^–4^

**Table 6:** Combinations of the four algorithm hyperparameters that combined the values in Tables 4 and 5 for a pre-trained CNN model

Number of experiments	Algorithm hyperparameters			
	Optimizer	MiniBatchSize	MaxEpochs	InitialLearnRate
1	adam	15	6	10^–6^
2	sgdm	25	9	10^–5^
3	adam	35	5	10^–4^
4	sgdm	10	8	10^–6^
5	adam	20	4	10^–5^
6	sgdm	30	7	10^–4^
7	adam	40	10	10^–4^

**Table 7:** Average correct rates and SDs in classifying chest CT images as COVID-19 positive or negative when VGG-19 and each algorithm hyperparameter combination in Table 6 were used in five independent experimental runs

Model# Experiment number	Dataset	Experimental runs						
		1	2	3	4	5	Average	SD
VGG-19#1	Training set	0.8252	0.8415	0.8448	0.8399	0.8399	0.8383	0.00757
	Validation set	0.8182	0.8182	0.8182	0.798	0.798	0.8101	0.01106
VGG-19#2	Training set	0.8578	0.8611	0.8709	0.8513	0.8497	0.8582	0.00851
	Validation set	0.8485	0.798	0.8283	0.8182	0.8182	0.8222	0.01835
VGG-19#3	Training set	0.5621	0.5784	0.6405	0.5931	0.4951	0.5738	0.05285
	Validation set	0.5859	0.6465	0.6566	0.6061	0.4949	0.5980	0.06447
VGG-19#4	Training set	0.8072	0.8056	0.7974	0.8039	0.8088	0.8046	0.00441
	Validation set	0.7879	0.7778	0.7879	0.7677	0.798	0.7839	0.01152
VGG-19#5	Training set	0.915	0.9036	0.915	0.915	0.8938	0.9085	0.00958
	Validation set	0.8384	0.8182	0.8081	0.8384	0.8182	0.8243	0.01355
VGG-19#6	Training set	0.9346	0.9265	0.9265	0.9346	0.9314	0.9307	0.00407
	Validation set	0.8485	0.8485	0.8485	0.8485	0.8586	0.8505	0.00452
VGG-19#7	Training set	0.8775	0.7026	0.7435	0.8693	0.8415	0.8069	0.07901
	Validation set	0.7374	0.7071	0.7475	0.8182	0.8081	0.7637	0.0477

**Fig. 2:**
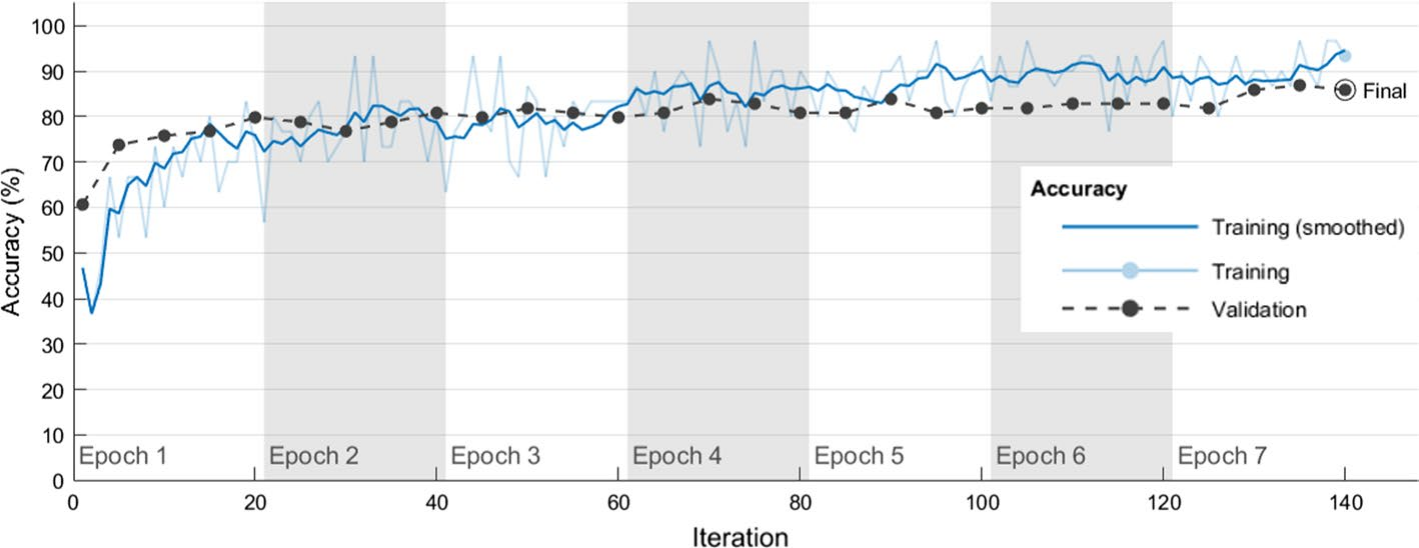
Progressive improvement in accuracy of VGG-19#6

According to the hyperparameter combination plan in Table 6, five independent experimental runs were performed for each hyperparameter combination. Table 7 shows the average correct rates and standard deviations (SDs) obtained by using each algorithm hyperparameter combination in Table 6 in five independent experimental runs when the VGG-19 was used to classify chest CT images as COVID-19 positive or negative in training and validation sets. The VGG-19#6 model had average correct rates of 93.07% and 85.05% in the training and validation sets, respectively. The VGG-19#6 also had small SDs of 0.00407 and 0.00452 in the training and validation sets, respectively. For classifying chest CT images as COVID-19 positive or negative, the best combination of the four algorithm hyperparameters in the VGG-19#6 model was Optimizer of ‘sgdm’, MiniBatchSize of 30, MaxEpochs of 7, and InitialLearnRate of 10^–4^. Figure 2 shows how model training progressively improved accuracy in VGG-19#6. Iterations per epoch were 20(≈612/30), which was the number of training images/MiniBatchSize. Maximum iterations were 140(= 20 × 7), which was iterations per epoch × MaxEpochs. The blue line shows the progressive improvement in accuracy for the training set, and the black line shows the progressive improvement in accuracy for the validation set.

**Fig. 3:**
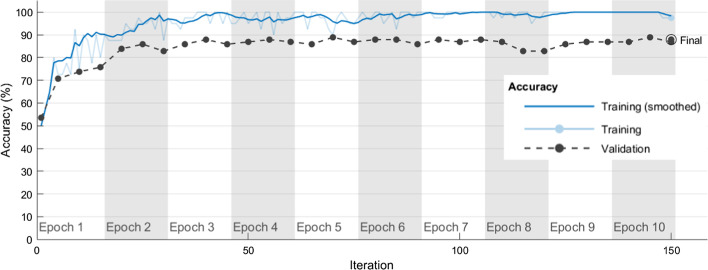
Progressive improvement in accuracy of Resnet-101#7

Table 8 shows the average correct rates and SDs obtained by using each algorithm hyperparameter combination in Table 6 in five independent experimental runs when the Resnet-101 model was used to classify chest CT images as COVID-19 positive or negative in training and validation sets. The Resnet-101#3 model had average correct rates of 98.10% and 87.88% in the training and validation sets, respectively. The Resnet-101#3 model also had small SDs of 0.00357 and 0 in the training and validation sets, respectively. In the Resnet-101#3 model, the hyperparameter combination with the high performance in classifying chest CT images as COVID-19 positive/negative was Optimizer of ‘adam’, MiniBatchSize of 35, MaxEpochs of 5, and InitialLearnRate of 10^–4^. The Resnet-101#7 model had average correct rates of 98.63% and 88.28% in the training and validation sets, respectively. The Resnet-101#7 model also had SDs of 0.00409 and 0.01835 in the training and validation sets, respectively. In the Resnet-101#7 model, the hyperparameter combination with the best performance in classifying chest CT images as COVID-19 positive/negative was Optimizer of ‘adam’, MiniBatchSize of 40, MaxEpochs of 10, and InitialLearnRate of 10^–4^. Figure 3 shows how model training progressively improved accuracy in Resnet-101#7. Iterations per epoch were 15(≈ 612/40), which was the number of training images/MiniBatchSize. Maximum iterations were 150(= 15 × 10), which was iterations per epoch × MaxEpochs. The blue line shows the progressive improvement in accuracy for the training set, and the black line shows the progressive improvement in accuracy for the validation set.

**Table 8:** Average correct rates and SDs in classifying chest CT images as COVID-19 positive/negative when Resnet-101 and each algorithm hyperparameter combination in Table 6 were used in five independent experimental runs

Model# experiment number	Dataset	Experimental runs						
		1	2	3	4	5	Average	SD
Resnet-101#1	Training set	0.7124	0.7059	0.7075	0.7042	0.7026	0.7065	0.00376
	Validation set	0.6061	0.596	0.6263	0.6162	0.6263	0.6142	0.01317
Resnet-101#2	Training set	0.7876	0.781	0.7876	0.7876	0.7892	0.7866	0.00321
	Validation set	0.6566	0.6566	0.6566	0.6566	0.6566	0.6566	0
Resnet-101#3	Training set	0.9804	0.9755	0.982	0.9853	0.982	0.9810	0.00357
	Validation set	0.8788	0.8788	0.8788	0.8788	0.8788	0.8788	0
Resnet-101#4	Training set	0.4951	0.5065	0.4951	0.4951	0.4951	0.4974	0.0051
	Validation set	0.5051	0.4848	0.5051	0.5051	0.5051	0.5010	0.00908
Resnet-101#5	Training set	0.9085	0.8987	0.9101	0.9101	0.9101	0.9075	0.00497
	Validation set	0.7879	0.7273	0.7879	0.798	0.798	0.7798	0.02979
Resnet-101#6	Training set	0.8366	0.8758	0.8317	0.835	0.835	0.8428	0.01852
	Validation set	0.7475	0.7677	0.7475	0.7475	0.7475	0.7515	0.00903
Resnet-101#7	Training set	0.9918	0.9869	0.9853	0.9804	0.9869	0.9863	0.00409
	Validation set	0.8687	0.899	0.8889	0.8586	0.899	0.8828	0.01835

**Fig. 4:**
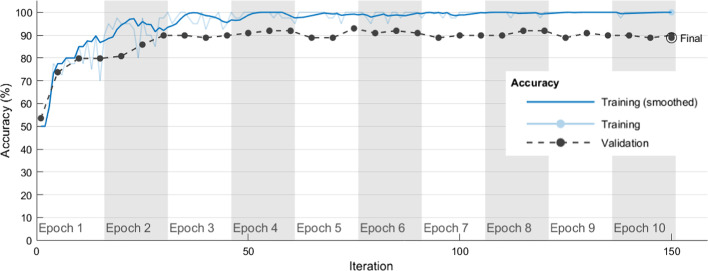
Progressive improvement in accuracy of DenseNet-201#7

Table 9 shows the average correct rates and SDs obtained for the training and validation sets when each algorithm hyperparameter combination in Table 6 was used in five independent experimental runs of the DenseNet-201 model to classify chest CT images as COVID-19 positive/negative. The DenseNet-201#3 model had average correct rates of 98.89% and 87.27% in the training and validation sets, respectively. This DenseNet-201#3 model also had small SDs of 0.00507 and 0.01532 in the training and validation sets, respectively. In the DenseNet-201#3 model, the hyperparameter combination with the high performance in classifying chest CT images as COVID-19 positive/negative was Optimizer of ‘adam’, MiniBatchSize of 35, MaxEpochs of 5, and InitialLearnRate of 10^–4^. The DenseNet-201#7 model had average correct rates of 99.28% and 88.49% in the training and validation sets, respectively. The DenseNet-201#7 model also had small SDs of 0.00469 and 0.0291 in the training and validation sets, respectively. In the DenseNet-201#7 model, the hyperparameter combination with the best performance in classifying chest CT images as COVID-19 positive/negative was Optimizer of ‘adam’, MiniBatchSize of 40, MaxEpochs of 10, and InitialLearnRate of 10^–4^. Figure 4 shows how model training progressively improved accuracy in DenseNet-201#7. Iterations per epoch were 15(≈612/40), which was the number of training images/MiniBatchSize. Maximum iterations were 150(= 15 × 10), which was iterations per epoch × MaxEpochs. The blue line shows the progressive improvement in accuracy for the training set, and the black line shows the progressive improvement in accuracy for the validation set.

**Table 9:** Average correct rates and SDs in classifying chest CT images as COVID-19 positive/negative when DenseNet-201 and each algorithm hyperparameter combination in Table 6 were used in five independent experimental runs

Model# experiment number	Dataset	Experimental runs						
		1	2	3	4	5	Average	SD
DenseNet-201#1	Training set	0.7484	0.7484	0.7484	0.7533	0.7402	0.7477	0.00472
	Validation set	0.6263	0.6263	0.6465	0.6364	0.6465	0.6364	0.0101
DenseNet-201#2	Training set	0.7745	0.7745	0.781	0.7859	0.7859	0.7804	0.00571
	Validation set	0.6667	0.6667	0.6667	0.6566	0.6768	0.6667	0.00714
DenseNet-201#3	Training set	0.9902	0.9853	0.9886	0.9967	0.9837	0.9889	0.00507
	Validation set	0.8788	0.8889	0.8788	0.8485	0.8687	0.8727	0.01532
DenseNet-201#4	Training set	0.598	0.598	0.6062	0.6078	0.6013	0.6023	0.00457
	Validation set	0.4949	0.4949	0.4848	0.4949	0.5051	0.4949	0.00718
DenseNet-201#5	Training set	0.9265	0.9281	0.9281	0.9248	0.9167	0.9248	0.00475
	Validation set	0.7677	0.7677	0.8182	0.7778	0.7879	0.7839	0.02094
DenseNet-201#6	Training set	0.9183	0.9183	0.9134	0.9118	0.9118	0.9147	0.00333
	Validation set	0.7677	0.7677	0.7677	0.8182	0.7576	0.7758	0.02411
DenseNet-201#7	Training set	0.9951	0.9967	0.9869	0.9886	0.9967	0.9928	0.00469
	Validation set	0.899	0.9293	0.8687	0.8586	0.8687	0.8849	0.0291

**Fig. 5:**
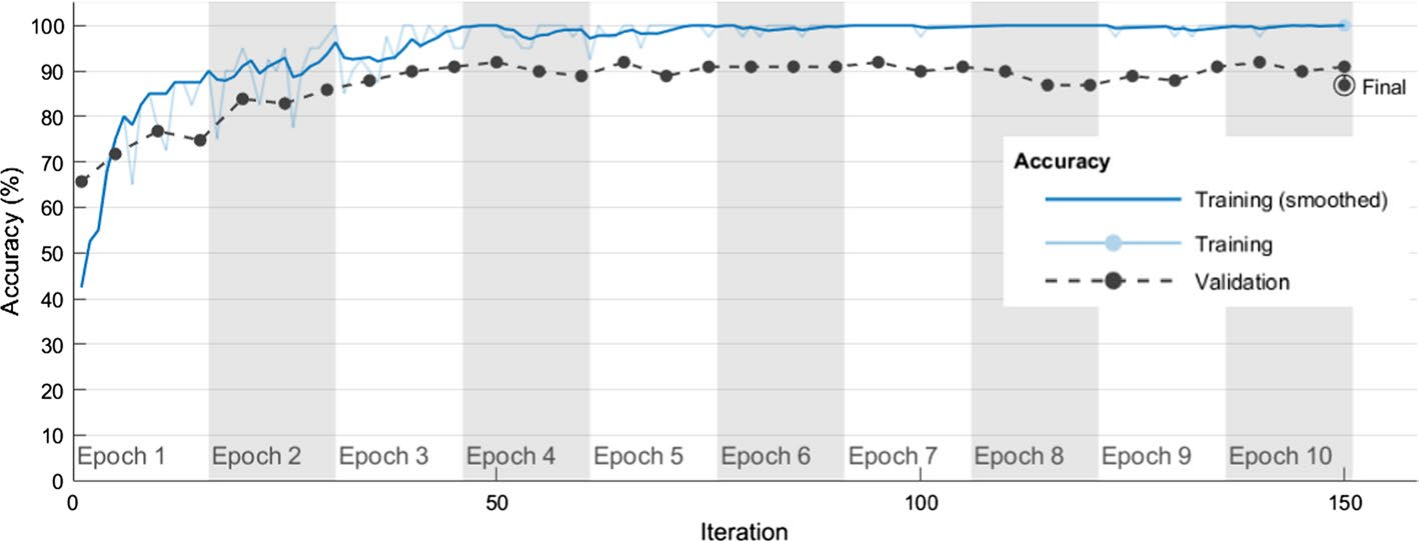
Progressive improvement in accuracy of Inception-v3#7

Table 10 shows the average correct rates and SDs obtained when each algorithm hyperparameter combination in Table 6 was used in five independent experimental runs of Inception-v3 to classify chest CT images as COVID-19 positive/negative in the training and validation sets. The Inception-v3#7 model had average correct rates of 98.89% and 86.67% in the training and validation sets, respectively. The Inception-v3#7 also had small SDs of 0.00355 and 0.01317 in the training and validation sets, respectively. In the Inception-v3#7 model, the hyperparameter combination with the best performance in classifying chest CT images as COVID-19 positive/negative was Optimizer of ‘adam’, MiniBatchSize of 40, MaxEpochs of 10, and InitialLearnRate of 10^–4^. Figure 5 shows how model training progressively improved accuracy in Inception-v3#7. Iterations per epoch were 15(≈ 612/40), which was the number of training images/MiniBatchSize. Maximum iterations were 150(= 15 × 10), which was iterations per epoch × MaxEpochs. The blue line shows the progressive improvement in accuracy for the training set, and the black line shows the progressive improvement in accuracy for the validation set.

**Table 10:** Average correct rates and SDs in classifying chest CT images as COVID-19 positive/negative when Inception-v3 and each algorithm hyperparameter combination in Table 6 were used in five independent experimental runs

Model# experiment number	Dataset	Experimental runs						
		1	2	3	4	5	Average	SD
Inception-v3#1	Training set	0.7418	0.7533	0.75	0.75	0.7484	0.7487	0.00425
	Validation set	0.7172	0.7475	0.7576	0.7374	0.7374	0.7394	0.01498
Inception-v3#2	Training set	0.6814	0.6699	0.683	0.6716	0.6716	0.6755	0.00618
	Validation set	0.7172	0.7071	0.7172	0.7071	0.7071	0.7111	0.00553
Inception-v3#3	Training set	0.9869	0.9869	0.9853	0.9869	0.982	0.9856	0.00213
	Validation set	0.8283	0.8485	0.8687	0.8485	0.8485	0.8485	0.01428
Inception-v3#4	Training set	0.5163	0.5196	0.5229	0.5147	0.5147	0.5176	0.00356
	Validation set	0.5556	0.5657	0.5859	0.5859	0.5859	0.5758	0.01428
Inception-v3#5	Training set	0.9118	0.8938	0.902	0.9003	0.9003	0.9016	0.00649
	Validation set	0.8182	0.8182	0.8283	0.7879	0.798	0.8101	0.0166
Inception-v3#6	Training set	0.8448	0.817	0.8301	0.8513	0.8513	0.8389	0.01499
	Validation set	0.798	0.7677	0.7677	0.7879	0.7879	0.7818	0.01355
Inception-v3#7	Training set	0.9869	0.9869	0.9918	0.9935	0.9853	0.9889	0.00355
	Validation set	0.8788	0.8788	0.8586	0.8687	0.8485	0.8667	0.01317

**Fig. 6:**
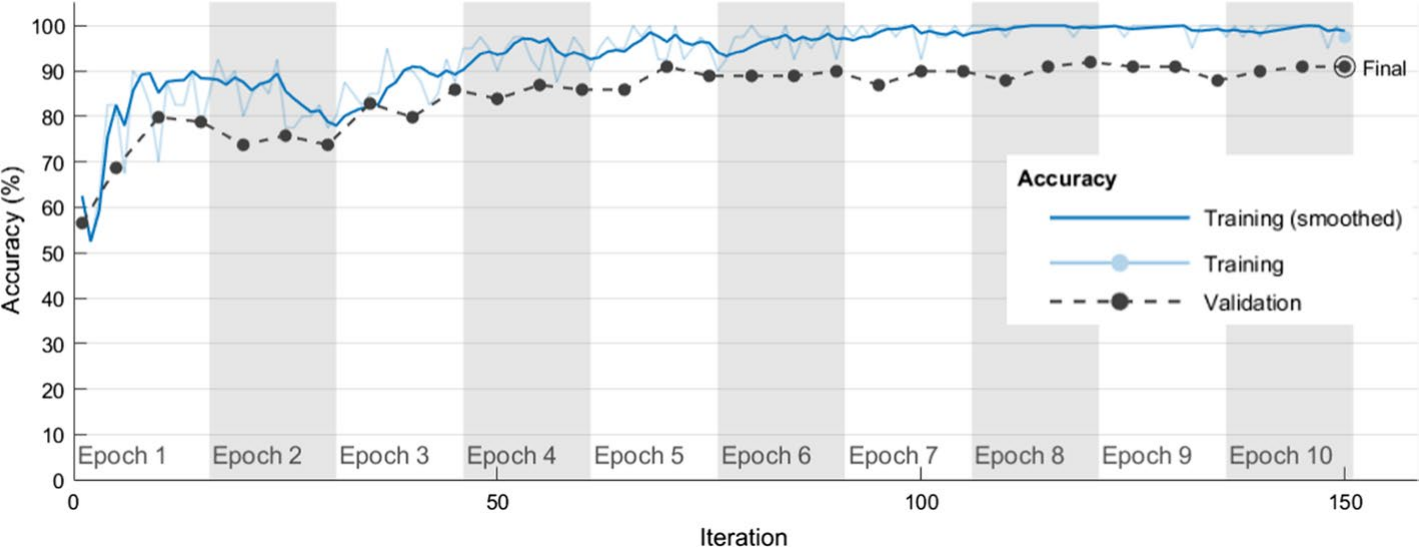
Progressive improvement in accuracy of Inception-ResNet-v2#7

Table 11 shows the average correct rates and SDs obtained when each algorithm hyperparameter combination in Table 6 was used in five independent experimental runs of Inception-ResNet-v2 to classify chest CT images as COVID-19 positive/negative in the training and validation sets. The Inception-ResNet-v2#3 model had average correct rates of 98.20% and 88.08% in the training and validation sets, respectively. The Inception-ResNet-v2#3 model also had small SDs of 0.00475 and 0.01807 in the training and validation sets, respectively. In the Inception-ResNet-v2#3 model, the hyperparameter combination with the high performance in classifying chest CT images as COVID-19 positive/negative was Optimizer of ‘adam’, MiniBatchSize of 35, MaxEpochs of 5, and InitialLearnRate of 10^–4^. The Inception-ResNet-v2#7 model had average correct rates of 98.66% and 90.91% in the training and validation sets, respectively. The Inception-ResNet-v2#7 model also had small SDs of 0.00647 and 0.0101 in the training and validation sets, respectively. The hyperparameter combination with the best performance in classifying chest CT images as COVID-19 positive/negative was Optimizer of ‘adam’, MiniBatchSize of 40, MaxEpochs of 10, and InitialLearnRate of 10^–4^. Figure 6 shows how model training progressively improved accuracy in Inception-ResNet-v2#7. Iterations per epoch were 15(≈612/40), which was the number of training images/MiniBatchSize. Maximum iterations were 150(= 15 × 10), which was iterations per epoch × MaxEpochs. The blue line shows the progressive improvement in accuracy for the training set, and the black line shows the progressive improvement in accuracy for the validation set.

**Table 11:** Average correct rates and SDs in classifying chest CT images as COVID-19 positive/negative when Inception-ResNet-v2 and each algorithm hyperparameter combination in Table 6 were used in five independent experimental runs

Model# experiment number	Dataset	Experimental runs						
		1	2	3	4	5	Average	SD
Inception-ResNet-v2#1	Training set	0.7092	0.7157	0.7271	0.7157	0.7141	0.7164	0.00657
	Validation set	0.6061	0.6061	0.6162	0.6061	0.6061	0.6081	0.00452
Inception-ResNet-v2#2	Training set	0.6471	0.6503	0.6454	0.6487	0.6307	0.6444	0.00789
	Validation set	0.5253	0.5051	0.5253	0.5051	0.5051	0.5132	0.01106
Inception-ResNet-v2#3	Training set	0.9755	0.9837	0.9853	0.9869	0.9788	0.9820	0.00475
	Validation set	0.899	0.8687	0.899	0.8788	0.8586	0.8808	0.01807
Inception-ResNet-v2#4	Training set	0.5114	0.5049	0.5098	0.5098	0.5033	0.5078	0.00352
	Validation set	0.4848	0.4848	0.4848	0.4848	0.5051	0.4889	0.00908
Inception-ResNet-v2#5	Training set	0.8971	0.902	0.9069	0.9003	0.9069	0.9026	0.00427
	Validation set	0.7677	0.7778	0.7475	0.7677	0.7879	0.7697	0.01498
Inception-ResNet-v2#6	Training set	0.7958	0.7876	0.8056	0.7876	0.781	0.7915	0.00946
	Validation set	0.6768	0.7172	0.697	0.7172	0.6566	0.6930	0.02634
Inception-ResNet-v2#7	Training set	0.9886	0.9755	0.9918	0.9869	0.9902	0.9866	0.00647
	Validation set	0.9192	0.9192	0.899	0.899	0.9091	0.9091	0.0101

According to the results of the thirty-five trained CNNs (shown in Tables 7, 8, 9, 10 and 11), the trained CNN shows that the average correct rates on the training set are high, and the average correct rates on the verification set are high. Table 12 shows the high classification accuracy obtained by the Resnet-101#3, Resnet-101#7, DenseNet-201#3, DenseNet-201#7, Inception-v3#7, Inception-ResNet-v2#3, and Inception-ResNet-v2#7 models. The SDs on the training set of the seven models are between 0.003 and 0.0065, indicating that the classification ability of the seven models is quite stable. The seven models for the validation set had average correct rates exceeding 0.86, though the average correct rate on the training set is 10% higher than that on the validation set. Therefore, the seven models were selected for inclusion in the ensemble model for classifying chest CT images as COVID-19 positive/negative.

**Table 12:** High accuracy of models obtained by Resnet-101#3, Resnet-101#7, DenseNet-201#3, DenseNet-201#7, Inception-v3#7, Inception-ResNet-v2#3, and Inception-ResNet-v2#7

Model	Dataset	Average	SD
Resnet-101#3	Training set	0.9810	0.00357
	Validation set	0.8788	0
Resnet-101#7	Training set	0.9863	0.00409
	Validation set	0.8828	0.01835
DenseNet-201#3	Training set	0.9889	0.00507
	Validation set	0.8727	0.01532
DenseNet-201#7	Training set	0.9928	0.00469
	Validation set	0.8849	0.0291
Inception-v3#7	Training set	0.9889	0.00355
	Validation set	0.8667	0.01317
Inception-ResNet-v2#3	Training set	0.9820	0.00475
	Validation set	0.8808	0.01807
Inception-ResNet-v2#7	Training set	0.9866	0.00647
	Validation set	0.9091	0.0101

The COVID19-CNN ensemble model, which combined Resnet-101#3, Resnet-101#7, DenseNet-201#3, DenseNet-201#7, Inception-v3#7, Inception-ResNet-v2#3, and Inception-ResNet-v2#7, used a majority voting strategy to classify chest CT images as COVID-19 positive/negative. An image classified as COVID-19 positive by most models was considered a COVID-19 image, and an image classified as COVID-19 negative by most models was considered a Normal image. The COVID19-CNN ensemble model aggregated the results of the majority voting strategy.

The accuracy metric was used to measure the performance of the Resnet-101#3, Resnet-101#7, DenseNet-201#3, DenseNet-201#7, Inception-v3#7, Inception-ResNet-v2#3, Inception-ResNet-v2#7, and COVID19-CNN models. Precision, recall, specificity, and F_1_-score were further used to validate classification performance. The results were depicted by creating a confusion matrix of the predicted labels versus the true labels for the respective classes. Table 13 shows the confusion matrix used for comparisons of COVID-19 positive and negative images in the Resnet-101#3, Resnet-101#7, DenseNet-201#3, DenseNet-201#7, Inception-v3#7, Inception-ResNet-v2#3, Inception-ResNet-v2#7, and COVID19-CNN models for the testing set.

**Table 13:** Confusion matrix for COVID-19 and Normal images obtained by the different trained CNN models and the COVID19-CNN ensemble model for the testing set

Model			True Labels	
			COVID-19	Normal
Resnet-101#3	Predicted	COVID-19	42	4
	Labels	Normal	4	41
Resnet-101#7	Predicted	COVID-19	43	3
	Labels	Normal	3	42
DenseNet-201#3	Predicted	COVID-19	43	2
	Labels	Normal	3	43
DenseNet-201#7	Predicted	COVID-19	45	7
	Labels	Normal	1	38
Inception-v3#7	Predicted	COVID-19	43	5
	Labels	Normal	3	40
Inception-ResNet-v2#3	Predicted	COVID-19	45	3
	Labels	Normal	1	42
Inception-ResNet-v2#7	Predicted	COVID-19	44	4
	Labels	Normal	2	41
COVID19-CNN	Predicted	COVID-19	45	2
	Labels	Normal	1	43

Based on the data in Tables 13 and 14 shows the classifier accuracy, precision, recall, specificity, and F_1_-score obtained by the different trained CNN models and COVID19-CNN ensemble models. When the testing set was used in the COVID19-CNN ensemble model, the accuracy was 0.967, which was superior to the accuracies obtained by the different trained CNN models. Other performance measures (i.e., precision, recall, specificity, and F_1_-score) obtained bythe COVID19-CNN ensemble model were higher than those obtained by the different trained CNN models. That is, the COVID19-CNN ensemble model had superior accuracy in classifying chest CT images as COVID-19 positive/negative.

**Table 14:** Classifier accuracy, precision, recall, specificity, and F_1_-score obtained by different trained CNN models and by the COVID19-CNN ensemble model for the testing set

Model	Accuracy	Precision	Recall	Specificity	F_1_-score
Resnet-101#3	0.912	0.913	0.913	0.911	0.913
Resnet-101#7	0.934	0.935	0.935	0.933	0.935
DenseNet-201#3	0.945	0.956	0.935	0.956	0.945
DenseNet-201#7	0.912	0.865	0.978	0.844	0.918
Inception-v3#7	0.912	0.896	0.935	0.889	0.915
Inception-ResNet-v2#3	0.956	0.938	0.978	0.933	0.957
Inception-ResNet-v2#7	0.934	0.917	0.957	0.911	0.936
COVID19-CNN	0.967	0.957	0.978	0.956	0.968

For the testing set of chest CT images from COVID-19 patients, the number of COVID-19 images ranged from 1 to 46, and the number of Normal images ranged from 47 to 91. Table 15 shows the numbers of images classified incorrectly by Resnet-101#3, Resnet-101#7, DenseNet-201#3, DenseNet-201#7, Inception-v3#7, Inception-ResNet-v2#3, Inception-ResNet-v2#7, and the COVID19-CNN ensemble model in the testing set. Eight classification errors occurred in the Resnet-101#3, DenseNet-201#7, and Inception-v3#7 models. Six classification errors occurred in the Resnet-101#7 and Inception-ResNet-v2#7 models. Five classification errors occurred in the DenseNet-201#3 models. Four classification errors occurred in the Inception-ResNet-v2#3 model. Image no. 46 was a COVID-19 image but was incorrectly classified as a Normal image by five models (i.e., Resnet-101#3, Resnet-101#7, DenseNet-201#3, Inception-v3#7, and Inception-ResNet-v2#3). Image no. 70 was a Normal image but was incorrectly classified as a COVID-19 image by seven models (i.e., Resnet-101#3, Resnet-101#7, DenseNet-201#3, DenseNet-201#7, Inception-v3#7, Inception-ResNet-v2#3, and Inception-ResNet-v2#7). Image no. 78 was a Normal image but was incorrectly classified as a COVID-19 image by four models (i.e., Resnet-101#3, Resnet-101#7, DenseNet-201#7, and Inception-v3#7). Therefore, the total number of classification errors made by the COVID19-CNN ensemble model using majority voting strategy was three. That is, the COVID19-CNN ensemble model had excellent capability in classifying chest CT images as COVID-19 positive/negative. Notably, image numbers 70 and 46 were incorrectly classified by seven and five models, respectively, and should be reviewed by a radiologist.

**Table 15:** Image numbers classified as wrong obtained by different trained CNN models and the COVID19-CNN ensemble model for the testing set

Model	Numbers
Resnet-101#3	11, 25, 34, 46, 64, 70, 78, 83
Resnet-101#7	25, 28, 46, 58, 70, 78
DenseNet-201#3	11, 28, 46, 64, 70
DenseNet-201#7	11, 60, 64, 70, 75, 78, 80, 81
Inception-v3#7	1, 29, 46, 52, 70, 77, 78, 90
Inception-ResNet-v2#3	46, 53, 60, 70
Inception-ResNet-v2#7	1, 28, 53, 61, 70, 83
COVID19-CNN	46, 70, 78

## Discussion

This study found that setting an appropriate combination of algorithm hyperparameters for a pre-trained CNN model was very important for accurately classifying chest CT images as COVID-19 positive or negative. In the VGG-19#6 model, for example, the appropriate combination of the four algorithm hyperparameters for classifying CT images was Optimizer of ‘sgdm’, MiniBatchSize of 30, MaxEpochs of 7, and InitialLearnRate of 10^–4^. In the Resnet-101#7, DenseNet-201#7, Inception-v3#7, and Inception-ResNet-v2#7 models, the appropriate combination was Optimizer of ‘adam’, MiniBatchSize of 40, MaxEpochs of 10, and InitialLearnRate of 10^–4^. In Resnet-101#3, DenseNet-201#3, and Inception-ResNet-v2#3, the appropriate combination was Optimizer of ‘adam’, MiniBatchSize of 35, MaxEpochs of 5, and InitialLearnRate of 10^–4^. Based on this study, it can be seen that a poor combination of algorithm hyperparameters for a pre-trained CNN model cannot get high accuracy in classifying chest CT images as COVID-19 positive/negative.

Although, from the novelty perspective, the contribution may be a relatively minor innovation, the COVID19-CNN ensemble model provided increased accuracy by applying a majority voting strategy and an appropriate combination of algorithm hyperparameters obtained by uniform experimental design can obtain high classification accuracy. Different trained CNN models had different results in classification of chest CT images as COVID-19 positive/negative, but the COVID19-CNN ensemble model used a majority voting mechanism to aggregate the results. Just like classifying chest CT images as COVID-19 positive/negative, the final classification results are determined by the opinions of most radiologists.

## Conclusions

This COVID19-CNN ensemble model proposed in this study effectively classified chest CT images as COVID-19 positive/negative. The main contributions of this study are the confirmation that the ensemble model provides increased accuracy by applying a majority voting strategy and the confirmation that an appropriate combination of algorithm hyperparameters can obtain high classification accuracy. Additionally, the image number of misclassifications can be found by the COVID19-CNN ensemble model when classifying chest CT images as COVID-19 positive/negative. When the COVID19-CNN ensemble model was used to classify CT images from the testing set of images as COVID-19 positive or negative, accuracy was 96.7%, which was superior to the accuracies obtained by Resnet-101#3 (91.2% accuracy), Resnet-101#7 (93.4% accuracy), DenseNet-201#3 (94.5% accuracy), DenseNet-201#7 (91.2% accuracy), Inception-v3#7 (91.2% accuracy), Inception-ResNet-v2#3 (95.6% accuracy), and Inception-ResNet-v2#7 (93.4% accuracy). Other performance measures obtained for the COVID19-CNN ensemble model (i.e., 95.7% precision, 97.8% recall, 95.6% specificity, and 96.8% F_1_-score) were also superior to those obtained by the different trained CNN models. That is, the COVID19-CNN ensemble model has excellent capability in classifying chest CT images as COVID-19 positive/negative.

## Methods

The research procedure was collecting data and processing chest CT images from COVID-19 patients, selecting multiple pre-trained CNN models for transfer learning, using UED to set algorithm hyperparameters for pre-trained CNN models, using multiple pre-trained CNN models to screen chest CT images for COVID-19, comparing classification performance among the trained CNN models, selecting the high accurate CNN models for further use in an ensemble model and, finally, comparing classification performance in the trained CNN models. The detailed steps were as follows.

### Collecting data and processing chest CT images from COVID-19 patients

The chest CT images from COVID-19 patients in Hu [11] were divided into a training set, a validation set, and a testing set. The training set had 612 chest CT images, including 309 images from COVID-19 patients and 303 images for the normal condition. The validation set had 99 chest CT images, including 50 images from COVID-19 patients and 49 images for the normal condition. The testing set used for network performance benchmarking contained 91 chest CT images, including 46 images from COVID-19 patients and 45 images for the normal condition. To maintain compatibility with the CNN-based architecture and the developed software, each CT image was processed as a 224 × 224 × 3 image or as a 299 × 299 × 3 image, where 3 is the number of color channels.

### Selecting multiple pre-trained CNN models for transfer learning

Transfer learning is a machine learning approach in which a model developed for a task is reused as the starting point for a model developed for another task. In transfer learning, a pre-trained CNN model is used to construct a predictive model. Thus, the first step is selecting a pre-trained CNN model from available models. The second step is reusing the pre-trained CNN model. The third step is tuning the pre-trained CNN model for a new task. Depending on the input–output pair data available for the new task, the researcher may consider further modification or refinement of the pre-trained CNN model. Transfer learning in a CNN model with pre-training is typically much faster than that in a CNN model without pre-training.

The widely used commercial software program Matlab R2019 by MathWorks has been validated as effective for pre-training CNN models for deep learning. Most pre-trained CNN models were trained with a subset of the ImageNet database [12] used in the ImageNet Large-Scale Visual Recognition Challenge [13]. After training on more than 1 million images, the pre-trained CNN models could classify images into 1000 object categories, e.g., keyboard, coffee mug, pencil, and various animals. The most important characteristics of pre-trained CNN models are network accuracy, speed, and size. Choosing a pre-trained network is generally a tradeoff between these characteristics. The classification accuracy on the ImageNet validation set is the most common way to measure the accuracy of networks trained on ImageNet. Networks that are accurate on ImageNet are also often accurate when you apply them to other natural image data sets using transfer learning or feature extraction.

According to comparing the ImageNet validation accuracy with the network accuracy, speed, and size, the pre-trained CNNs used to classify chest CT images were VGG-19, Resnet-101, DenseNet-201, Inception-v3, and Inception-ResNet-v2.

The VGG-19 [14], Resnet-101 [15], and DenseNet-201 [16] CNNs have 19 layers, 101 layers, and 201 layers, respectively, and have been trained on more than 1 million images from the ImageNet database. As a result, these CNNs have learned rich feature representations for a wide range of images and can classify images into 1000 object categories. The image input size for these CNNs is 224 × 224 × 3.

The 48-layer Inception-v3 [17] and the 164-layer Inception-ResNet-v2 [18] CNNs have been trained on more than 1 million images from the ImageNet database and can classify images into 1000 object categories. The image input size for these CNNs is 299 × 299 × 3.

### Using UED to design algorithm hyperparameters for pre-trained CNN models

The UED method developed by Wang and Fang [19–21] used space filling designs to construct a set of experimental points uniformly scattered in a continuous design parameter space. Because UED only considers uniform dispersion and not comparable orderliness, UED minimizes the number of experiments needed to acquire all available information.

Selecting appropriate algorithm hyperparameters for a pre-trained CNN model was essential for accurate screening of chest CT images for COVID-19. In this study, the algorithm hyperparameters for a pre-trained CNN model were Optimizer, MiniBatchSize, MaxEpochs, and InitialLearnRate. The combinations of algorithm hyperparameters obtained by UED were used in a pre-trained CNN model to classify chest CT images as COVID-19 positive/negative.

### Screening chest CT images for COVID-19 by multiple pre-trained CNN models

**Fig. 7:**
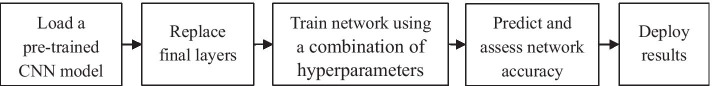
Flowchart of transfer learning procedure used in the CNN model

To fine-tune a pre-trained CNN model, transfer learning is often faster and easier than constructing and training a new CNN model for a new task. Although a pre-trained CNN model has already learned a rich set of image features, it can be fine-tuned to learn features specific to a new dataset (i.e., chest CT images from COVID-19 patients in this study). Since a pre-trained CNN model can learn to extract a different feature set, the final CNN model is often more accurate. The starting point for fine tuning deeper layers of pre-trained CNN models used for transfer learning (i.e., VGG-19, Resnet-101, DenseNet-201, Inception-v3, and Inception-ResNet-v2) was training the networks with a new dataset of chest CT images from COVID-19 patients. Figure 7 is a flowchart of the transfer learning procedure used in the CNN model.

### Comparing classification performance among different trained CNN models

In this study, five independent runs of VGG-19, Resnet-101, DenseNet-201, Inception-v3, and Inception-ResNet-v2 were performed to classify chest CT images as COVID-19 positive or negative by using an algorithm hyperparameter combination obtained by UED. The results recorded for the training set and the validation set included (1) accuracy in each run of the experiment, (2) average accuracy in five independent runs, and (3) standard deviation in accuracy in five independent runs. Accuracy was defined as the proportion of true positive or true negative results for a population.

### Selecting the trained CNN models with the high accuracy for use in a COVID19-CNN ensemble model and comparing the classification performance of the ensemble model with that of other trained CNN models

The high accurate CNN models after training with VGG-19, Resnet-101, DenseNet-201, Inception-v3, and Inception-ResNet-v2 were selected for use in an ensemble model for classifying images in the testing set of chest CT images as COVID-19 positive or negative.

The classification performance of the different trained CNN models was compared in terms of accuracy, precision, recall (i.e., sensitivity), specificity, and F_1_-score values. Precision was assessed by positive predictive value (number of true positives over number of true positives plus number of false positives). Recall (sensitivity) was assessed by true positive rate (number of true positives over the number of true positives plus the number of false negatives). Specificity was measured by true negative rate (number of true negatives over the number of false positives plus the number of true negatives). The F_1_-score, a function of precision and recall, was used to measure prediction accuracy when classes were very imbalanced. In information retrieval, precision is a measure of the relevance of results while recall is a measure of the number of truly relevant results returned. The formula for F_1_-score is 2 × (precision × recall)/(precision + recall).

## Data Availability

All data obtained and analyzed during this study are included in this article. Chest CT images of COVID-19: https://github.com/KevinHuRunWen/COVID-19/blob/master/data.zip.

## Abbreviations

COVID-19: Coronavirus disease 2019
CT: Computed tomography
UED: Uniform experimental design
CNN: Convolutional neural network
